# Not all workplace bullying perpetrators are alike: health, personality, and occupational profiles of perpetrator-only and target-perpetrator employees

**DOI:** 10.3389/fpsyg.2026.1890622

**Published:** 2026-09-23

**Authors:** Junchen Sun, Gülüm Özer, Jordi Escartín

**Affiliations:** 1 Department of Psychological Medicine, Institute of Psychiatry, Psychology & Neuroscience, King's College London, London, United Kingdom; 2 Department of Social Psychology and Quantitative Psychology, Universitat de Barcelona, Barcelona, Spain

**Keywords:** workplace bullying, conservation of resources theory, Social Dominance Theory, target-perpetrator, perpetration, personality traits, psychological distress

## Abstract

**Background:**

Research on workplace bullying (WB) has primarily focused on targets, whereas those who engage in bullying have often been treated as a homogeneous group. It remains unclear whether employees who only enact bullying (perpetrator-only) and those who both enact and experience bullying (target-perpetrator) represent distinct forms of perpetration-related involvement.

**Objective:**

The present study examined whether perpetrator-only and target-perpetrator groups could be differentiated based on their health-related, personality, and occupational profiles.

**Methods:**

Cross-sectional Wave 1 data from a three-wave longitudinal study were analyzed. A total of 2,359 employees were classified into four WB identity groups based on behavioral reports of exposure to and enactment of negative acts: perpetrator-only (*n* = 51), target-perpetrator (*n* = 149), target-only (*n* = 576), and not-involved (*n* = 1,583). Group comparisons and adjusted binary logistic regression models were used to examine differences across the four groups, with particular emphasis on the perpetrator-only and target-perpetrator groups.

**Results:**

The target-perpetrator group showed the most adverse overall descriptive profile. Psychological distress and chronic psychological conditions were disproportionately concentrated in this group. In adjusted models, psychological distress was robustly associated with greater odds of membership in the target-perpetrator group rather than the perpetrator-only group. Neuroticism showed a positive association in the conventional adjusted model, but the adjusted bootstrap estimate was not significant. No other health-related or personality variables independently differentiated the two groups.

**Conclusion:**

This study addresses a major gap in WB research by demonstrating that employees involved in perpetration are heterogeneous and that perpetrator-only and target-perpetrator groups show meaningfully different profiles. Distress-related vulnerability was particularly concentrated among employees occupying the dual role. These findings support more theoretically informed and role-sensitive approaches to WB prevention, assessment, and intervention.

## Introduction

Workplace bullying (WB) is increasingly recognized as a significant global issue, impacting individuals and organizations worldwide. It is a form of interpersonal misconduct characterized by persistent and harmful behaviors, occurring repeatedly over time within an organization (Einarsen et al., 2020). These negative behaviors include gossiping (Crothers et al., 2009), verbal abuse and persistent criticism (Einarsen, 2000), and also social exclusion and professional sabotage (Serenko, 2020).

Prevalence estimates underscore the scope of the problem. Approximately 15% of employees report experiencing negative behaviors at work (Nielsen and Einarsen, 2018). Across Europe, 10%−20% of employees report occasional exposure, while higher rates have been reported in the USA, ranging from 38 to 71% (León-Pérez et al., 2021). However, perpetration rates are less frequently reported. A systematic review of 50 studies found that self-labeling approaches yield mean perpetration rates around 4%, whereas behavioral assessments yield around 8% (Özer and Escartín, 2023). In addition, the prevalence of target-perpetrators, who both experience and enact bullying behaviors, has been reported to be 37.2% irrespective of frequency (Özer and Escartín, 2024).

Bullying has substantial implications for both individuals and organizations. Research has linked WB exposure with psychological distress (Einarsen and Nielsen, 2015) and physical health complaints (Glambek et al., 2018). At the organizational level, WB has been associated with actual turnover, changing employer, and broader organizational dysfunction (Glambek et al., 2015). However, despite the well-established impact of WB, existing research has focused mainly on targets, with comparatively less attention devoted to those involved in perpetration.

### Health states, personality, and WB perpetration

Within the comparatively limited literature on perpetration, the psychological and physical health characteristics of perpetrators remain insufficiently understood. Evidence indicates that those who enact bullying may report elevated psychological distress and physical health symptoms (Balducci et al., 2022), particularly in negative organizational contexts (Özer et al., 2022). However, these findings are difficult to interpret when all perpetrators are treated as a single group. The distinction between perpetrator-only and target-perpetrator groups is especially important because the latter occupy a dual role in WB, being both targets and perpetrators of negative acts.

Target-perpetrators may therefore carry a compounded burden involving the strain of victimization, the impact of retaliatory or defensive behavior, and the psychological cost of escalating conflict. Although evidence remains limited, previous findings suggest that target-perpetrators may show poorer physical health than other groups involved in WB (Brotheridge et al., 2012). Health-related indicators may therefore be useful for differentiating perpetration-related profiles. Because the present study is cross-sectional, these health indicators are conceptualized as correlates of WB role involvement rather than definitive antecedents or consequences.

The health-related variables examined in the present study represented four complementary aspects of employee functioning: current psychological strain, somatic manifestations of strain, long-term health burden, and health-risk behaviors. Psychological distress provides a relatively proximal indicator of current emotional strain and impaired functioning. Physical symptoms capture health complaints, such as sleep difficulties, headaches, and dizziness, which may accompany sustained occupational stress even in the absence of a diagnosed condition. Chronic conditions represent a more enduring health burden that may constrain employees' capacity to perform and cope with interpersonal conflict and workplace demands. Alcohol and tobacco consumption were included as modifiable health-risk behaviors that may co-occur with psychosocial work stress and affect longer-term health and functioning; previous research has specifically linked workplace psychosocial stressors to alcohol use (Nielsen et al., 2018). These indicators capture different processes and timescales and were therefore not treated as interchangeable measures of a single adverse-health construct.

In addition to health-related characteristics, personality traits may help account for variation in different forms of bullying perpetration. Personality traits shape how individuals perceive, process and interact with others (Wilson and Nagy, 2017). Previous research has linked WB perpetration to higher levels of Machiavellianism, narcissism, psychoticism, and aggression (Linton and Power, 2013), as well as to lower levels of agreeableness, fairness, and sincerity (Dåderman and Ragnestål-Impola, 2019). More recent work has also reported positive associations of perpetration with narcissism and sadism, as well as negative associations with agreeableness (Fernández Del Río et al., 2021).

Gender and perceived gender composition of the workplace were included as demographic variables because WB may reflect gendered patterns of interaction and power. Previous research has identified predominantly same-gender targeting and perpetration, potentially reflecting occupational gender segregation and differences in interaction opportunities (Lee and Brotheridge, 2011). Examining gender composition across WB role groups therefore helps contextualize whether group differences are associated with role membership or with broader gendered workplace structures.

### Research gaps and implications

Most studies, however, treat perpetrators as a homogeneous group. This approach risks obscuring meaningful role-specific differences, particularly between the perpetrator-only group and the target-perpetrator group. Without explicit role differentiation, findings may produce overly generalized profiles and intervention recommendations.

A key empirical gap is the limited direct comparison between perpetrator-only and target-perpetrator groups. It therefore remains unclear whether health-related and personality characteristics associated with perpetrators reflect perpetration itself, concurrent victimization, or their combination.

Importantly, the target-perpetrator group should not be understood solely as a subtype of perpetrator or target. Within the present role framework, these employees report both exposure to and enactment of negative acts and are therefore relevant to both perpetrator- and target-focused research. Longitudinal evidence suggests that exposure to and enactment of WB can be reciprocal, with employees potentially moving between or simultaneously occupying these roles as interpersonal conflict escalates (Rodríguez-Muñoz et al., 2015; Vranjes et al., 2023). However, the target-perpetrator group may fall between two established research traditions. Perpetrator-focused research may obscure this group by aggregating all employees who enact negative acts, whereas target-focused research may overlook the possibility that some targets also engage in such acts. Their health-related, personality, and sociodemographic characteristics should therefore not automatically be interpreted as fixed perpetrator predispositions, but should also be considered in relation to victimization-related strain, resource loss, and coping demands within adverse working conditions.

Although the term ‘provocative victim' has been used in earlier research (Matthiesen and Einarsen, 2007), the present study uses ‘target-perpetrator' because it provides a more neutral and behaviorally descriptive label for employees who report both exposure to and enactment of negative acts. It also avoids implying that these employees caused or were partly responsible for their own victimization. Moreover, because the present analysis is cross-sectional and cannot establish whether victimization or perpetration occurred first, the term ‘target-perpetrator' more accurately denotes the co-occurrence of the two reported roles without imposing a causal sequence.

A related theoretical gap is that Conservation of Resources theory and Social Dominance Theory have rarely been integrated to explain heterogeneity within perpetration-related involvement. The present study addresses these gaps by distinguishing the perpetrator-only group from the target-perpetrator group and drawing on both theories as complementary perspectives. In doing so, it moves beyond the assumption that perpetrators constitute a homogeneous group and provides a basis for more role-sensitive organizational responses.

By comparing demographic characteristics, health-related indicators, and personality traits across WB roles, the present study sought to determine whether perpetrator-only and target-perpetrator involvement represent meaningfully distinct profiles, potentially reflecting different patterns of strain or emotional reactivity. This study therefore had two aims: first, to examine whether distinct forms of perpetration-related involvement could be identified; and second, to assess whether the perpetrator-only and target-perpetrator groups differed in their sociodemographic, health-related, and personality profiles. The study also considered whether these differences might have implications for role-sensitive organizational responses. To achieve these aims, the study had three objectives: (1) to classify participants into four WB groups; (2) to compare these groups across sociodemographic characteristics, health-related indicators, and personality traits; and (3) to examine whether health-related and personality variables were associated with the odds of membership in the target-perpetrator group rather than the perpetrator-only group.

### Theoretical framework: distinguishing perpetrator-only and target-perpetrator groups

To account for heterogeneity in WB perpetration, this section draws on two complementary theoretical perspectives. We propose that perpetrator-only and target-perpetrator involvement may reflect role-specific configurations in which resource protection, status maintenance or restoration, and responses to interpersonal threat are combined in different ways.

Conservation of Resources theory proposes that people strive to obtain, retain, and protect valued resources, and that actual or threatened resource loss generates stress and impairs functioning (Hobfoll, 1989). Among the 74 resources identified within Conservation of Resources theory (Hobfoll and Lilly, 1993), health is a core personal resource because it enables survival, daily functioning, and access to other valued resources. Good health supports individuals' capacity to work, maintain social relationships, cope with stress, and invest effort and energy to gain further resources, whereas loss of health represents a particularly powerful form of resource loss. Such loss not only produces distress but also depletes time, energy, finances, and social support, increasing vulnerability to further losses and creating downward loss spirals. Health is therefore both a foundational resource and a key outcome of successful resource conservation and protection.

For the target-perpetrator group, the combination of experiencing victimization and engaging in WB may reflect and intensify resource depletion. Psychological distress, physical symptoms, chronic conditions and unhealthy lifestyle behaviors may therefore signal reduced resources and impaired self-regulation.

Although target-perpetrators may face a broader and potentially cumulative pattern of resource loss because they report both exposure to and enactment of bullying within the same reference period, this is a matter of degree rather than a process unique to this group. Perpetrator-only involvement is not necessarily resource-neutral. Occupational status, reputation and social support may constitute valued resources. Threats to these resources, together with continuing effort required to maintain or defend them, may generate distress even in the absence of concurrent victimization. The target-perpetrator group, however, may face additional layer of victimization-related resource threat. Their involvement is therefore expected to show a stronger association with current health-related distress, whereas perpetrator-only involvement may be relatively more consistent with instrumental, role-based or dominance-oriented motives. Accordingly, employees reporting a greater health-related burden are expected to have higher odds of belonging to the target-perpetrator group than the perpetrator-only group. Among the examined indicators, psychological distress is expected to show the clearest association with membership in the target-perpetrator group rather than the perpetrator-only group because it is most closely aligned with current emotional strain.

H1a: Higher levels of psychological distress will be associated with greater odds of membership in the target-perpetrator group rather than the perpetrator-only group.

H1b: Higher levels of physical symptoms will be associated with greater odds of membership in the target-perpetrator group rather than the perpetrator-only group.

H1c: The presence of chronic disease will be associated with greater odds of membership in the target-perpetrator group rather than the perpetrator-only group.

H1d: Higher levels of alcohol and tobacco use will be associated with greater odds of membership in the target-perpetrator group rather than the perpetrator-only group.

Social Dominance Theory proposes that social hierarchies are maintained through individual behaviors, institutional practices, and legitimizing beliefs that sustain unequal relations (Sidanius and Pratto, 1999). In workplace settings, bullying others can be conceptualized as a hierarchy-enhancing behavior through which individuals intimidate, devalue, or exclude others to maintain control, protect status, or preserve occupational advantage. Perpetrator-only involvement may therefore be more consistent with status-maintenance, control, or instrumental motives than with current emotional distress. On this account, the perpetrator-only group may show relatively lower emotional reactivity and, in some cases, appear more socially controlled or agreeable.

Social Dominance Theory may also help explain target-perpetrator involvement. When victimization is perceived as a threat to control or relative standing, negative behaviors may partly serve to restore a threatened position or reassert control. Perpetration in the target-perpetrator group may therefore reflect both interpersonal strain and a hierarchy- or status-restoring response to perceived threat. Higher neuroticism could heighten sensitivity to interpersonal threat, lower agreeableness could reduce empathy, cooperation, and willingness to repair conflict, and lower conscientiousness could weaken self-regulation and adherence to organizational norms. These traits may be particularly relevant when perpetration is embedded in an ongoing cycle of victimization, perceived injustice, and retaliation. As a result, the target-perpetrator group may be expected to report higher levels of neuroticism-driven reactions and to be less characterized by strategic, goal-directed aggression than the perpetrator-only group. Accordingly, the present study tests whether higher neuroticism, lower agreeableness, and lower conscientiousness differentiate the perpetrator-only group from the target-perpetrator group.

H2a: Higher neuroticism will be associated with greater odds of membership in the target-perpetrator group rather than the perpetrator-only group.

H2b: Lower agreeableness will be associated with greater odds of membership in the target-perpetrator group rather than the perpetrator-only group.

H2c: Lower conscientiousness will be associated with greater odds of membership in the target-perpetrator group rather than the perpetrator-only group.

## Materials and methods

### Procedure and sample

The present study reports a secondary, cross-sectional analysis of Wave 1 data collected as part of a broader three-wave longitudinal study (Özer et al., 2022). The parent study was designed to examine whether health conditions predict subsequent WB perpetration. For the current analysis, variables were retained only if they were directly relevant to the study aims, hypotheses, and the conceptual framework informed by Conservation of Resources theory and Social Dominance Theory. Wave 1 data were collected between March and June 2021 via an anonymous online questionnaire. Although subsequent waves were completed, only Wave 1 data were included in the present analysis. Recruitment was conducted in Spain and Türkiye with the support of two academics in psychology and organizational psychology. Their students were invited to recruit employees working at least 8 h per week and received one additional course credit for recruiting ten eligible respondents. The survey was also advertised in three languages through Facebook, LinkedIn, and Instagram. All participants were informed about confidentiality, voluntary participation and withdrawal rights before providing consent. Ethical approval was granted by the Universitat de Barcelona (IRB00003099).

Of 3,663 respondents, 1,074 were excluded because they worked fewer than 8 h per week (*n* = 478), did not provide consent (*n* = 544), or submitted duplicate responses (*n* = 52), resulting in an eligible sample of 2,589 participants. Of these, 230 were excluded because their WB role status could not be determined. The remaining 2,359 participants formed the main analytic sample for WB identity classification and group-based analyses. The average age was 34.40 years (*SD* = 12.24); 57.60% of participants were female and 42.40% were male. Most participants resided in Türkiye (57.69%) and Spain (37.81%). Participants represented a range of industries, most commonly services (15.1%), education (11.4%), health (9.3%), manufacturing (8.7%), and wholesale and retail trade (5.9%). Their average organizational tenure was 7.3 years (*SD* = 8.9), and they worked an average of 5.1 days per week (*SD* = 0.86).

### Instruments and variables

#### Measures of WB

##### Target of WB

The 4-item Workplace Bullying Scale-Reduced (EAPA-T-R; Escartín et al., 2017) assessed WB from the target's perspective (e.g., “My correspondence, telephone calls or work assignments have been controlled or blocked”) over the past 6 months on a 1 (Never) to 7 (very frequently/more than once a week) scale. The original Cronbach's α was 0.74 and α in the present study was 0.70.

##### Perpetrator of WB

The 4-item modified perpetrator version of the Workplace Bullying Scale-Reduced (EAPA-T-R; Özer et al., 2022) assessed enactment of WB over the past 6 months (e.g., “I controlled or blocked correspondence, telephone calls or work assignments of others”), rated 1–7. The current α was 0.76.

#### Measures of health-related conditions

##### Psychological distress

The 4-item scale from Restubog et al. (2011) captured symptoms such as feeling fearful, restless, worthless, or panicked, over the past month, rated 1 (Never) to 7 (Always). Cronbach's α was 0.81 in both the original and current studies.

##### Physical symptoms

The 13-item Physical Symptoms Inventory (Duffy et al., 2019) assessed complaints like nausea, trouble sleeping or headaches in the past month, on a 7-point Likert scale ranging from 1 (Never) to 7 (Always). Previous studies have reported internal consistency reliability values ranging from 0.81 to 0.94; in the present study, Cronbach's α was 0.86.

##### Chronic disease

Chronic diseases were measured using a checklist adapted from Eurostat (2020). Participants were asked: “Have you ever been informed by a physician that you have or have had one or more of the following conditions? (Multiple responses were allowed)”. Each condition (e.g., asthma, diabetes) was recorded dichotomously (0 = no, 1 = yes). Participants could also report additional conditions via an open-ended item (“Please state if you have any other illnesses, conditions or treatments”).

For analysis, data from the predefined list and open-ended responses were combined and recoded into six binary categories (0 = not reported, 1 = reported): Psychological condition (e.g., depression, bipolar), Thyroid Condition (e.g., hypothyroidism, hyperthyroidism), Rheumatic Condition (e.g., arthritis, rheumatism), Migraine Condition (e.g., chronic migraine, cluster headache), Digestive Condition (e.g., irritable bowel syndrome, other gastrointestinal issues), and Heart Condition (e.g., myocardial infarction, coronary heart disease).

##### Alcohol and tobacco consumption

Alcohol and tobacco consumption were measured using two single items adapted from Eurostat (2020), assessing the frequency of alcohol use and smoking (including tobacco products, heated tobacco and e-cigarettes) over the past 6 months using a 6-point Likert-type scale ranging from “I don't drink/smoke” to “Every day.”

#### Measures of personality

The 20-item Mini-International Personality Item Pool (Mini-IPIP; Donnellan et al., 2006) assessed Conscientiousness, Extraversion, Agreeableness, Neuroticism and Openness to experience (a construct capturing intellectual curiosity, abstract thinking and imagination) on a 1 (Completely False) to 7 (Completely True) scale (e.g., “I have a vivid imagination”). Cronbach's α values for each dimension were as follows: Extraversion (original α = 0.77, current α = 0.52), Agreeableness (original α = 0.70, current α = 0.67), Conscientiousness (original α = 0.69, current α = 0.72), Neuroticism (original α = 0.68, current α = 0.51), and Openness to experience (original α = 0.65, current α = 0.61). Although the internal consistency for Extraversion (α = 0.52) and Neuroticism (α = 0.51) were lower than typically desired, this is not unexpected for ultra-short measures with only four items per dimension. Cronbach's α is highly sensitive to the number of items (Pallant, 2020), and the original scale authors noted that the Mini-IPIP often yields modest reliability coefficients due to its brevity. McDonald's omega was also computed as a supplementary reliability estimate. The reliability estimates were as follows: Extraversion ω = 0.55, Agreeableness ω = 0.62, Conscientiousness ω = 0.73, Neuroticism ω = 0.47, Openness to experience ω = 0.58. Conscientiousness showed acceptable internal consistency, Agreeableness showed modest internal consistency, and Extraversion, Neuroticism, and Openness to experience showed relatively low internal consistency. Accordingly, findings involving Extraversion, Neuroticism, and Openness to Experience, particularly those relating to the hypothesis concerning Neuroticism, should be interpreted with caution.

##### Demographic information

Demographic information collected at Wave 1 included variables such as age, gender, nationality, supervisory position, and perceived workplace gender composition.

##### Classification of WB groups

Participants were classified into four WB role groups based on self-reported exposure to and enactment of negative acts. The four-group structure was informed by previous WB studies that distinguished perpetrators, targets, dual-role target-perpetrators, and employees not involved in either role (Matthiesen and Einarsen, 2007; Glasø et al., 2009). In these studies, respondents who confirmed that they had bullied others were classified as perpetrators, while those reporting both perpetration and victimization were classified as dual-role or provocative victims. The present study retained this role-based framework but operationalised involvement using behaviorally worded measures of specific negative acts rather than relying solely on direct self-labeling.

Although repetition and persistence are central to most definitions of WB, the frequency threshold used to identify involvement remains debated. One relatively stringent definition requires exposure to at least one negative act on a weekly basis over a 6-month period (Einarsen et al., 2020). However, frequency alone may not fully capture the impact of WB, as even occasional exposure has been associated with poorer self-reported mental health (Hansen et al., 2011). Accordingly, Lange et al. (2019) adopted a broader behavioral approach in which reported exposure to any negative act was treated as indicating bullying involvement. The four WB groups were examined to establish the broader role context, while the main theoretical and hypothesis-focused analyses concerned the distinction between perpetrator-only and target-perpetrator groups. In line with this approach, we used both scale means and individual item thresholds to classify participants into four distinct groups based on their responses:

##### Perpetrator-only group

These individuals reported enacting WB behaviors without being exposed to bullying themselves. Participants were classified as perpetrators if their average score on the perpetrator scale was ≥2, indicating they engaged in bullying at least a few times in the past 6 months, or if any single item score was ≥4, indicating that bullying occurred occasionally, monthly, or more frequently.

##### Target-only group

These individuals reported exposure to WB behaviors without engaging in bullying themselves. Participants were classified as targets if their average score on the target scale was ≥ 2, indicating they experienced bullying at least a few times over the past 6 months, or if any single item score was ≥4, indicating that they were bullied occasionally, monthly, or more frequently.

##### Target-perpetrator group (dual role)

The target-perpetrator group comprised participants who reported both being targeted and enacting bullying behaviors. They met the cut-off criterion on both the target and perpetration scales.

##### Not-involved group

Participants who did not meet the cut-off on either scale and gave no other indication of bullying exposure or enactment.

The primary dual-threshold classification was intended to capture broader behavioral involvement over the past 6-month reference period rather than only high-frequency cases. The scale-mean criterion allowed the identification of involvement reflected across the full set of negative acts, whereas the single-item criterion captured a more concentrated occurrence of a particular negative act. Because the resulting role classification may depend on this operational decision, an alternative, stricter frequency criterion was examined in a sensitivity analysis.

### Data analysis

Missing data were not imputed. Complete responses to all eight WB items were not required in every case. Participants with item-level missingness were retained when their observed responses were sufficient to classify them unambiguously under the prespecified rule. For each four-item scale, target or perpetrator status was assigned when the fixed-denominator score, the sum of the observed item responses divided by four, was ≥2, or when at least one observed item was rated ≥4. If neither criterion was met and one or more items were missing, status was treated as indeterminate rather than as non-involved. Participants were assigned to one of the four WB role groups only when both target and perpetrator status could be determined. Descriptive statistics and correlations were based on available cases, whereas group-comparison analyses excluded cases missing the variables required for each test, and logistic regression analyses used complete cases for all variables included in the respective model. Consequently, sample sizes varied across analyses because of item-level missing data and differences in the variables included in each analysis. Group differences among the perpetrator-only, target-perpetrator, target-only and not-involved groups were examined using Kruskal–Wallis tests for continuous variables and chi-square tests for categorical variables. When significant group differences were found, Bonferroni-adjusted *post hoc* comparisons were conducted to examine pairwise differences among all four groups.

The hypothesis-focused analyses then compared the target-perpetrator and perpetrator-only groups using two hierarchical binary logistic regression models. Group membership was coded as 1 = target-perpetrator and 0 = perpetrator-only. In the health-related analysis, psychological distress, physical symptoms, chronic psychological condition, alcohol consumption, and tobacco consumption were entered in Model 1, and age, gender, and supervisory role were added in Model 2. In the personality analysis, the personality traits were entered in Model 1, and age, gender, and supervisory role were added in Model 2. To address concerns about the relatively small perpetrator-only cell in the direct comparison with target-perpetrator, the two logistic regression models were additionally examined using non-parametric bootstrap resampling. Bootstrap analyses were based on 5,000 stratified bootstrap samples, with resampling stratified by target-perpetrator vs. perpetrator-only status. This procedure preserved the group distribution across resamples and was used to assess the stability and precision of the regression coefficients, odds ratios, and confidence intervals.

Group-comparison tests were used to examine whether the WB identity groups differed on demographic, health-related, and personality variables, whereas regression analyses were used to examine whether health-related and personality variables predicted membership in the target-perpetrator group vs. the perpetrator-only group after accounting for demographic factors.

To examine whether the findings depended on the primary dual-threshold classification rule, the four-group classification and both logistic regression analyses comparing target-perpetrator with perpetrator-only membership were repeated using a stricter criterion. In this sensitivity analysis, participants were classified as targets or perpetrators only if they reported at least one relevant negative act occurring at least weekly, defined as any single item score of ≥6. Participants were classified as target-perpetrators only if they met this weekly-occurrence criterion on both the target and perpetration scales. Participants with partial item-level missingness were retained only when an observed item already met this threshold. When no observed item was rated ≥6 but one or more items were missing, weekly-frequency status was treated as indeterminate because the missing response could have altered the classification. Consequently, two additional participants were excluded from the sensitivity classification compared with the primary classification.

Missing-data patterns were examined using Little's MCAR test. To examine whether missingness on the target and perpetration scales was related to observed participant characteristics, participants with complete and incomplete scales were compared using chi-square tests for categorical variables and Mann-Whitney U tests for continuous variables. Missing WB items were not imputed because role classification required sufficient observed item-level information to determine target and perpetrator status unambiguously. Participants whose role status could not be determined were excluded from role-based analyses, and available-case analysis was used for the remaining analyses. All analyses were conducted using IBM SPSS Statistics, Version 32.0.0.0 (134) (IBM Corp., Armonk, NY, USA).

## Results

Of the 2,359 participants, 576 (24.42%) were classified as target-only, 51 (2.16%) as perpetrator-only, 149 (6.32%) as target-perpetrator, and 1,583 (67.10%) as not-involved. Little's MCAR test was significant, χ^2^ (546) = 842.53, *p* < 0.001, indicating that the missing-data pattern was inconsistent with MCAR. After correction for multiple comparisons, missingness on both the target and perpetration scales remained significantly associated with openness to experience scores and was also associated with chronic psychological condition status, suggesting that missingness was related to observed participant characteristics. The results of the present study are organized according to the study objectives. Descriptive statistics for the four WB groups are presented first, followed by group comparisons in sociodemographic characteristics, health-related indicators, and personality traits. The subsequent analyses focus on the perpetrator-only and target-perpetrator groups to identify the health-related and personality variables associated with membership in these two perpetration-related groups. Finally, bootstrap analyses are reported to assess the robustness of the key regression findings.

### WB role distribution and correlates

Normality was examined prior to the analyses. As expected, given the typically skewed nature of WB data, all continuous variables significantly deviated from normality (*p* < 0.001). Despite significant deviations from normality, Pearson correlations were used because the analyses involved composite scale scores, the sample size was large, and Pearson correlations are generally robust to non-normality in large samples. Table 1 presents descriptive statistics and intercorrelations for the main study variables. Nationality and gender composition were not included in the hypothesis-focused regression models because they were not central to the study hypotheses and was not significantly associated with target or perpetration scores in preliminary analyses. Extraversion was retained in the personality model for completeness, although no specific hypothesis was proposed for this trait. Although many correlations were statistically significant, most were small. Several small-to-moderate correlations linked the perpetration score with psychological distress, physical symptoms, chronic conditions and personality traits. In general, WB scores were positively associated with health-related problems and negatively associated with adaptive personality traits such as agreeableness and conscientiousness.

**Table 1:** Descriptives and correlations.

Variable	*N*	Mean	*SD*	1	2	3	4	5	6	7	8	9	10	11	12	13	14	15	16
1. Age	2,340	34.40	12.24	—															
2. Gender	2,339	0.42	0.49	0.02	—														
3. Supervisory Role	2,326	0.28	0.45	0.22^**^	0.17^**^	—													
4. Psychological Distress	2,431	2.40	1.10	−0.15^**^	−0.12^**^	−0.00	—												
5. Physical Symptoms	2,406	2.36	0.86	−0.22^**^	−0.19^**^	−0.05^*^	0.54^**^	—											
6. Psychological Condition	2,588	0.13	0.33	−0.01	−0.07^**^	−0.04	0.22^**^	0.23^**^	—										
7. Migraine Condition	2,588	0.01	0.09	−0.00	−0.03	0.01	0.04	0.05^*^	0.03	—									
8. Heart Condition	2,588	0.02	0.13	0.07^**^	0.02	0.02	0.01	0.04	0.06^**^	0.05^*^	—								
9. Alcohol Consumption	2,407	2.87	1.59	0.10^**^	0.07^**^	0.11^**^	0.03	−0.04^*^	0.05^*^	0.01	0.00	—							
10. Tobacco Consumption	2,404	2.86	2.24	−0.13^**^	0.12^**^	0.03	0.06^**^	0.11^**^	0.06^**^	0.02	0.02	0.15^**^	—						
11. Agreeableness	2,364	5.52	1.05	0.14^**^	−0.21^**^	0.01	−0.05^*^	−0.02	−0.03	−0.01	−0.02	0.04^*^	−0.06^**^	—					
12. Conscientiousness	2,365	5.43	1.17	0.17^**^	−0.04	0.06^**^	−0.11^**^	−0.10^**^	−0.08^**^	0.01	0.03	−0.13^**^	−0.03	0.20^**^	—				
13. Neuroticism	2,362	3.74	1.15	−0.15^**^	−0.24^**^	−0.08^**^	0.40^**^	0.37^**^	0.21^**^	0.02	0.01	0.02	0.02	−0.03	−0.23^**^	—			
14. Openness to experience	2,365	5.02	1.16	−0.16^**^	0.02	0.00	−0.01	−0.00	−0.02	0.00	−0.01	0.06^**^	0.03	0.23^**^	0.08^**^	−0.09^**^	—		
15. Target Score	2,386	1.66	0.91	−0.07^**^	−0.06^**^	0.01	0.45^**^	0.35^**^	0.16^**^	0.05^*^	0.00	0.03	0.08^**^	−0.11^**^	−0.11^**^	0.20^**^	−0.02	—	
16. Perpetration Score	2,376	1.22	0.57	−0.10^**^	0.01	0.08^**^	0.21^**^	0.20^**^	0.10^**^	0.01	0.04^*^	0.06^**^	0.10^**^	−0.16^**^	−0.18^**^	0.11^**^	−0.11^**^	0.42^**^	—

Descriptive statistics and correlations were based on available cases from the eligible sample of 2,589 participants. Ns vary because of item-level missing data.

Gender: 0 = female, 1 = male; Supervisory position: 0 = not in supervisory position, 1 = in supervisory position; Variables 6–8: 0 = not having related chronic condition, 1 = have related chronic condition.

^*^p < 0.05.

^**^p < 0.01.

### Health and personality differences across WB roles

Kruskal–Wallis tests showed significant group differences in age, psychological distress, physical symptoms, alcohol consumption, tobacco consumption and personality traits including agreeableness, conscientiousness, neuroticism, and openness to experience (all *p* < 0.05; Table 2).

**Table 2:** Results of Kruskal–Wallis test examining the effect of WB identity on demographic, health-related variables, and personality variables.

Variable	*N*	**Mean (*****SD***)				Kruskal–Wallis *H* (df = 3)
		Perpetrator-only	Target-perpetrator	Target-only	Not-involved	
Age	2,315	31.74 (11.61)	31.07 (11.09)	33.79 (11.46)	35.00 (12.58)	15.56^**^
Psychological Distress	2,358	2.41 (1.05)	3.23 (1.35)	2.94 (1.18)	2.12 (0.91)	318.27^***^
Physical Symptoms	2,355	2.47 (0.77)	2.93 (1.05)	2.66 (0.86)	2.18 (0.76)	197.26^***^
Alcohol Consumption	2,358	3.35 (1.70)	3.03 (1.58)	2.92 (1.55)	2.83 (1.60)	8.12^*^
Tobacco Consumption	2,357	3.96 (2.33)	3.50 (2.25)	2.99 (2.27)	2.72 (2.21)	38.16^***^
Agreeableness	2,339	5.41 (1.22)	4.93 (1.22)	5.49 (1.05)	5.60 (1.00)	44.03^***^
Conscientiousness	2,339	5.32 (1.24)	4.72 (1.31)	5.41 (1.13)	5.50 (1.15)	50.59^***^
Neuroticism	2,338	3.76 (1.25)	4.29 (1.04)	4.00 (1.12)	3.60 (1.14)	88.56^***^
Openness to experience	2,340	4.82 (1.25)	4.63 (1.26)	5.10 (1.19)	5.04 (1.13)	20.51^***^

^*^p < 0.05.

^**^p < 0.01.

^***^p < 0.001.

For health-related variables, psychological distress showed the most pronounced group difference, *H*_(3)_ = 318.27, *p* < 0.001. Descriptively, the target-perpetrator group reported the highest level of psychological distress (*M* = 3.23), suggesting that individuals who occupied both target and perpetrator roles experienced particularly elevated distress compared with other WB groups. Physical symptoms also differed significantly across groups, indicating that WB identity was associated not only with psychological wellbeing but also with self-reported physical health.

The personality variables also showed a consistent pattern across WB identity groups. Descriptively, the target-perpetrator group displayed the least favorable profile among the assessed personality traits, with the lowest levels of agreeableness (*M* = 4.93), conscientiousness (*M* = 4.72), and openness to experience (*M* = 4.63), as well as the highest level of neuroticism (*M* = 4.29). In contrast, the target-only and perpetrator-only groups were more similar to each other and to the not-involved group on several personality traits. Overall, these descriptive results identify the target-perpetrator group as the most differentiated group, particularly in psychological distress and personality measures.

Kruskal–Wallis tests indicated significant differences across WB identity groups; however, these omnibus tests do not specify which groups differ from one to another. Therefore, Bonferroni-adjusted *post-hoc* Dunn's comparisons were conducted to identify specific group differences. The results of these comparisons are presented in Table 3.

**Table 3:** Bonferroni-adjusted pairwise Dunn's tests following Kruskal–Wallis tests.

Comparisons	Age											
	*Z*	*SE*	*p*									
Demographic												
Target-only × perpetrator-only	−1.53	98.54	0.756									
Target-only × target-perpetrator	−2.52	62.86	0.070									
Target-only × not-involved	−1.30	32.76	1.000									
Perpetrator-only × target-perpetrator	−0.07	109.95	1.000									
Perpetrator-only × not-involved	−2.02	95.97	0.263									
Target-perpetrator × not-involved	3.43	58.75	0.004^**^									
**Comparisons**	**Psychological Distress**			**Physical Symptoms**			**Alcohol Consumption**			**Tobacco Consumption**		
	***z***	***SE***	***p***	***z***	***SE***	***p***	***z***	***SE***	***p***	***z***	***SE***	***p***
Health-Related Factor												
Target-only × perpetrator-only	−3.30	99.10	0.006^**^	−1.33	99.28	1.000	−1.87	96.95	0.367	−3.05	89.98	0.014^*^
Target-only × target-perpetrator	−1.73	62.34	0.505	−2.19	62.63	0.172	−0.73	60.99	1.000	−2.71	56.76	0.041^*^
Target-only × not-involved	−15.69	33.01	<0.001^***^	−11.89	33.08	<0.001^***^	−1.25	32.29	1.000	−2.78	29.97	0.032^*^
Perpetrator-only × target-perpetrator	−3.95	110.04	<0.001^***^	−2.44	110.34	0.088	−1.27	107.66	1.000	−1.21	100.00	1.000
Perpetrator-only × not-involved	−1.98	96.50	0.284	−2.70	96.68	0.042^*^	−2.35	94.41	0.113	−4.08	87.62	<0.001^***^
Target-perpetrator × not-involved	10.76	58.13	<0.001^***^	9.08	58.42	<0.001^***^	1.49	56.87	0.817	4.48	52.94	<0.001^***^
**Comparisons**	**Agreeableness**			**Conscientiousness**			**Neuroticism**			**Openness to experience**		
	***z***	***SE***	***p***	***z***	***SE***	***p***	***z***	***SE***	***p***	***z***	***SE***	***p***
Personality Factor												
Target-only × perpetrator-only	−0.18	99.30	1.000	−0.46	99.35	1.000	−1.51	99.35	0.786	−1.48	99.43	0.836
Target-only × target-perpetrator	−4.98	62.44	<0.001^***^	−5.60	62.31	<0.001^***^	−3.05	62.31	0.014^*^	−4.42	62.36	<0.001^***^
Target-only × not-involved	−2.10	32.90	0.217	−1.83	32.92	0.407	−7.02	32.94	<0.001^***^	−1.57	32.94	0.704
Perpetrator-only × target-perpetrator	−2.65	110.33	0.048^*^	−2.75	110.29	0.036^*^	−3.09	110.28	0.012^*^	−1.17	110.38	1.000
Perpetrator-only × not-involved	−0.90	96.72	1.000	−1.09	96.77	1.000	−0.84	96.76	1.000	−0.99	96.85	1.000
Target-perpetrator × not-involved	6.52	58.25	<0.001^***^	7.04	58.10	<0.001^***^	7.25	58.10	<0.001^***^	3.85	58.15	0.001^***^

*p*-values have been Bonferroni-adjusted for multiple comparisons.

^*^p < 0.05.

^**^p < 0.01.

^***^p < 0.001.

*Post hoc* comparisons indicated that the target-perpetrator group was significantly younger than the not-involved group. No other age comparisons were significant. Therefore, age appears to represent a limited contextual group difference rather than a primary factor distinguishing WB identity groups.

For health-related indicators, psychological distress showed a clear involvement-related pattern. Both target-only and target-perpetrator participants reported significantly higher psychological distress than not-involved participants. In addition, both groups reported significantly higher psychological distress than the perpetrator-only group. Although the target-perpetrator group had the highest mean level of psychological distress descriptively, they did not differ significantly from target-only participants. The perpetrator-only group also did not differ significantly from not-involved participants on psychological distress.

For physical symptoms, all three WB-involved groups reported significantly more physical symptoms than the not-involved group. However, target-only, perpetrator-only, and target-perpetrator groups did not differ significantly from one another. Thus, physical symptoms appeared to distinguish WB-involved participants from not-involved participants, rather than clearly separating the different WB-involved groups.

For tobacco consumption, all three WB-involved groups reported significantly higher tobacco consumption than the not-involved group. Perpetrator-only and target-perpetrator groups also reported significantly higher tobacco consumption than the target-only group. However, perpetrator-only and target-perpetrator groups did not differ significantly from each other. Although the perpetrator-only group had the highest mean tobacco consumption descriptively, this difference was not statistically significant relative to the target-perpetrator group. For alcohol consumption, although the omnibus Kruskal–Wallis test was significant, none of the Bonferroni-adjusted pairwise comparisons reached significance. Therefore, alcohol-related differences should be interpreted cautiously.

For personality traits, target-perpetrators showed the most distinct profile. They scored significantly lower on agreeableness and conscientiousness than target-only, perpetrator-only, and not-involved groups. They also scored significantly higher on neuroticism than all three other groups. In addition, target-only participants scored significantly higher on neuroticism than not-involved participants. However, perpetrator-only group did not differ significantly from either target-only or not-involved participants on neuroticism. For openness to experience, target-perpetrator participants scored significantly lower than target-only and not-involved participants. However, target-perpetrator did not differ significantly from perpetrator-only group on openness to experience. The perpetrator-only group also did not differ significantly from target-only or not-involved participants. Therefore, openness to experience primarily distinguished target-perpetrator from the target-only and not-involved groups, but it did not clearly distinguish the perpetrator-only group from the other groups.

Comparisons involving the single-role groups were generally limited. Target-only and perpetrator-only groups differed significantly only in psychological distress and tobacco consumption: target-only participants reported higher psychological distress, whereas the perpetrator-only group reported higher tobacco consumption. They did not differ significantly in age, physical symptoms, alcohol consumption, or any personality trait. Compared with the target-perpetrator group, the perpetrator-only group reported lower psychological distress, higher agreeableness and conscientiousness, and lower neuroticism. However, they did not differ from target-perpetrator in age, physical symptoms, alcohol consumption, tobacco consumption, or openness to experience. Compared with the not-involved group, the perpetrator-only group differed only in physical symptoms and tobacco consumption, but not in psychological distress or personality traits.

Overall, the *post hoc* analyses suggest that the target-perpetrator group was the most clearly differentiated WB identity group, particularly in relation to psychological distress and personality traits. However, the pattern was not uniform across all variables. Psychological distress distinguished target-only and target-perpetrator participants from both not-involved and perpetrator-only groups, whereas physical symptoms distinguished all WB-involved groups from the not-involved group. Tobacco consumption was elevated across all WB-involved groups, with perpetrator-only and target-perpetrator groups reporting higher levels than target-only participants. Personality differences were most pronounced for the target-perpetrator group, especially for agreeableness, conscientiousness, and neuroticism. These results suggest that dual involvement in WB is associated with the most pronounced psychological and personality-related differences, whereas single-role involvement is associated with a more limited and variable pattern of differences.

Chi-square analyses were conducted for categorical variables (Table 4). Significant associations were found for gender, supervisory role, and chronic psychological condition, while all other categorical variables (Country Living, Perceived Workplace Gender Composition, Thyroid Condition, Rheumatic Condition, Migraine Condition, Digestive Condition, Heart Condition) showed no significant differences among WB groups.

**Table 4:** Bonferroni-adjusted pairwise chi-square comparisons between WB identity and gender, supervisory role, and chronic psychological condition.

Comparison	Variable														
	Gender					Supervisory role					Chronic psychological condition				
	Female		Male		*X^2^(v)*	No		Yes		*X^2^(v)*	No		Yes		*X^2^(v)*
	Observed	Expected	Observed	Expected		Observed	Expected	Observed	Expected		Observed	Expected	Observed	Expected	
Target-only × perpetrator- Only					16.37^***^ (0.16)					7.85^*^ (0.11)					0.48 (0.03)
Target-only	358	344.00	209	222.40		418	409.60	144	152.40		475	476.80	101	99.20	
Perpetrator-only	17	30.40	33	19.60		28	36.40	22	13.60		44	42.20	7	8.80	
Target-only × target-perpetrator					1.40 (0.04)					3.21 (0.07)					8.49^*^ (0.11)
Target-only	358	351.90	209	215.10		418	409.50	144	152.50		475	462.40	101	113.60	
Target-perpetrator	82	88.10	60	53.90		95	103.50	47	38.50		107	119.60	42	29.40	
Target-only × not-involved					7.19^*^ (0.06)					0.58 (0.02)					19.77^***^ (0.10)
Target-only	358	331.10	209	235.90		418	411.20	144	150.80		475	505.00	101	71.00	
Not-involved	881	907.90	674	647.10		1,125	1,131.80	422	415.20		1,418	1,388.00	165	195.00	
Perpetrator-only × target-perpetrator					8.35^*^ (0.21)					1.91 (0.10)					4.30 (0.15)
Perpetrator-only	17	25.80	33	24.20		28	32.00	22	18.00		44	38.50	7	12.50	
Target-perpetrator	82	73.20	60	68.80		95	91.00	47	51.00		107	112.50	42	36.50	
Perpetrator-only × not-involved					10.09^**^ (0.08)					6.75 (0.07)					0.57 (0.02)
Perpetrator-only	17	28.00	33	22.00		28	36.10	22	13.90		44	45.60	7	5.40	
Not-involved	881	870.00	674	685.00		1,125	1,116.90	422	430.10		1,418	1,416.40	165	166.60	
Target-perpetrator × not-involved					0.06 (0.01)					2.20 (0.04)					40.84^***^ (0.15)
Target-perpetrator	82	80.60	60	61.40		95	102.60	47	39.40		107	131.20	42	17.80	
Not-involved	881	882.40	674	672.60		1,125	1,117.40	422	429.60		1,418	1,393.80	165	189.20	

*p*-values have been Bonferroni-adjusted for multiple comparisons. Values in parentheses are Cramér's V.

Country Living, Thyroid Condition, Rheumatic Condition, Migraine Condition, Digestive Condition and Heart Condition were not significantly associated with WB groups (all *p* > 0.05).

^*^p < 0.05.

^**^p < 0.01.

^***^p < 0.001.

For gender, the target-only group had a notably higher proportion of females compared to both the perpetrator-only group, χ^2^ (1, *N* = 617) = 16.37, *p* < 0.001, Cramér's *V* = 0.16, and the not-involved group, χ^2^ (1, *N* = 2,122) = 7.19, *p* < 0.05, Cramér's *V* = 0.06. The perpetrator-only group showed a higher proportion of males in comparison to both the target-perpetrator group, χ^2^ (1, *N* = 192) = 8.35, *p* < 0.05, Cramér's *V* = 0.21, and the not-involved group, χ^2^ (1, *N* = 1,605) = 10.09, *p* < 0.01, Cramér's *V* = 0.08. No significant gender differences were found in the comparisons of other WB identity groups.

For supervisory role, the pattern was more limited. The only significant pairwise comparison was between the target-only and perpetrator-only groups, χ^2^ (1, *N* = 612) = 7.85, *p* < 0.05, Cramér's *V* = 0.11. The perpetrator-only group showed a higher-than-expected proportion of supervisory roles, whereas target-only participants showed a lower-than-expected proportion. No other pairwise comparisons for supervisory role were significant after Bonferroni adjustment.

Chronic psychological condition showed the clearest categorical health-related pattern. The target-perpetrator group had a higher-than-expected prevalence of chronic psychological condition when compared with target-only participants, χ^2^ (1, *N* = 725) = 8.49, *p* < 0.05, Cramér's *V* = 0.11, and especially when compared with not-involved participants, χ^2^ (1, *N* = 1,732) = 40.84, *p* < 0.001, Cramér's *V* = 0.15. Target-only participants also showed a higher-than-expected prevalence of chronic psychological condition than not-involved participants, χ^2^ (1, *N* = 2,159) = 19.77, *p* < 0.001, Cramér's *V* = 0.10. However, perpetrator-only group did not differ significantly from target-only, target-perpetrator, or not-involved groups in chronic psychological condition status.

Overall, the *post hoc* chi-square comparisons suggest that categorical differences across WB identity groups were most evident for gender, supervisory role, and chronic psychological condition. The target-perpetrator group was particularly distinguished by elevated prevalence of chronic psychological condition, supporting the view that dual-role involvement is associated with heightened vulnerability. In contrast, perpetrator-only group was primarily differentiated by gender distribution and supervisory-role status rather than health-related indicators. These findings suggest that different forms of WB involvement may reflect distinct underlying patterns of vulnerability and organizational positioning.

### Factors distinguishing target-perpetrator from perpetrator-only membership

Hierarchical binary regression analyses were conducted to examine whether health-related conditions (Table 5) and personality traits (Table 6) distinguished target-perpetrator from perpetrator-only membership. Extraversion was not significantly associated with target or perpetration scores in preliminary analyses, but it was retained in the personality model for completeness. Because only chronic psychological condition showed a significant categorical association with WB identity groups and other chronic disease categories had low prevalence or insufficient case numbers, chronic psychological condition was used as the chronic-disease indicator in the logistic regression model. Target-perpetrator status was coded as 1 and perpetrator-only status as 0. Model 1 included the main predictors, while Model 2 additionally controlled for age, gender and supervisory role.

**Table 5:** Logistic regression models for health-related conditions.

Predictor	Model 1			Model 2		
	*B*	OR	95% CI for OR	*B*	OR	95% CI for OR
Psychological Distress	0.54	1.72	[1.16, 2.54]^**^	0.52	1.68	[1.11, 2.53]^*^
Physical Symptoms	0.20	1.22	[0.84, 1.78]	0.16	1.17	[0.78, 1.75]
Psychological Condition	0.25	1.29	[0.94, 1.76]	0.22	1.25	[0.91, 1.72]
Alcohol consumption	−0.22	0.80	[0.57, 1.14]	−0.13	0.88	[0.61, 1.26]
Tobacco consumption	−0.30	0.74	[0.52, 1.06]	−0.27	0.77	[0.53, 1.10]
Age				0.12	1.13	[0.76, 1.68]
Gender				−0.30	0.74	[0.51, 1.08]
Supervisory role				−0.11	0.89	[0.64, 1.26]

^*^p < 0.05.

^**^p < 0.01.

Migraine condition and heart condition were excluded because of insufficient case numbers.

**Table 6:** Logistic regression models for personality traits.

Predictor	Model 1			Model 2		
	*B*	OR	95% CI for OR	*B*	OR	95% CI for OR
Extraversion	−0.23	0.80	[0.56, 1.14]	−0.24	0.79	[0.54, 1.14]
Agreeableness	−0.32	0.73	[0.52, 1.01]	−0.35	0.71	[0.50, 1.00]
Conscientiousness	−0.29	0.75	[0.54, 1.03]	−0.31	0.73	[0.52, 1.02]
Neuroticism	0.54	1.71	[1.19, 2.46]^**^	0.42	1.52	[1.03, 2.25]^*^
Openness to experience	−0.06	0.94	[0.68, 1.30]	−0.06	0.94	[0.67, 1.31]
Age				0.15	1.16	[0.77, 1.75]
Gender				−0.44	0.65	[0.44, 0.96]^*^
Supervisory role				−0.01	0.99	[0.70, 1.40]

Agreeableness did not reach conventional statistical significance in either model, although the estimate was in the expected direction: Model 1, *p* = 0.057; Model 2, *p* = 0.051.

^*^p < 0.05.

^**^p < 0.01.

For health-related variables, psychological distress was associated with greater odds of target-perpetrator membership in Model 1, *B* = 0.54, OR = 1.72, 95% CI [1.16, 2.54], *p* < 0.01. This association remained significant after controlling for demographic and work-related covariates in Model 2, *B* = 0.52, OR = 1.68, 95% CI [1.11, 2.53], *p* < 0.05. Thus, higher psychological distress was associated with greater odds of belonging to the target-perpetrator group rather than the perpetrator-only group. Physical symptoms, chronic psychological condition, alcohol consumption and tobacco consumption were not significant predictors in either model. Therefore, H1b, H1c and H1d were not supported. Age, gender, and supervisory role were also not significant in the adjusted model. Migraine condition and Heart condition were excluded from the model due to insufficient case numbers.

For personality traits, neuroticism was significantly associated with greater odds of target-perpetrator group membership in Model 1, *B* = 0.54, OR = 1.71, 95% CI [1.19, 2.46], *p* < 0.01. After controlling for age, gender, and supervisory role, neuroticism remained significant, *B* = 0.42, OR = 1.52, 95% CI [1.03, 2.25], *p* < 0.05. Thus, higher neuroticism was associated with increased odds of belonging to the target-perpetrator rather than the perpetrator-only group. Agreeableness, extraversion, conscientiousness, and openness to experience were not significant predictors. Although the coefficient for agreeableness was in the expected direction and approached conventional significance, it did not reach the 0.05 threshold. Therefore, H2b and H2c were not supported. In the adjusted personality model, gender was also significant, *B* = −0.44, OR = 0.65, 95% CI [0.44, 0.96], *p* < 0.05, whereas age and supervisory role were not significant. Figure 1 presents the adjusted odds ratios and 95% confidence intervals from the health-related and personality models. The confidence intervals for the remaining focal predictors included the null value of 1.

**Figure 1:**
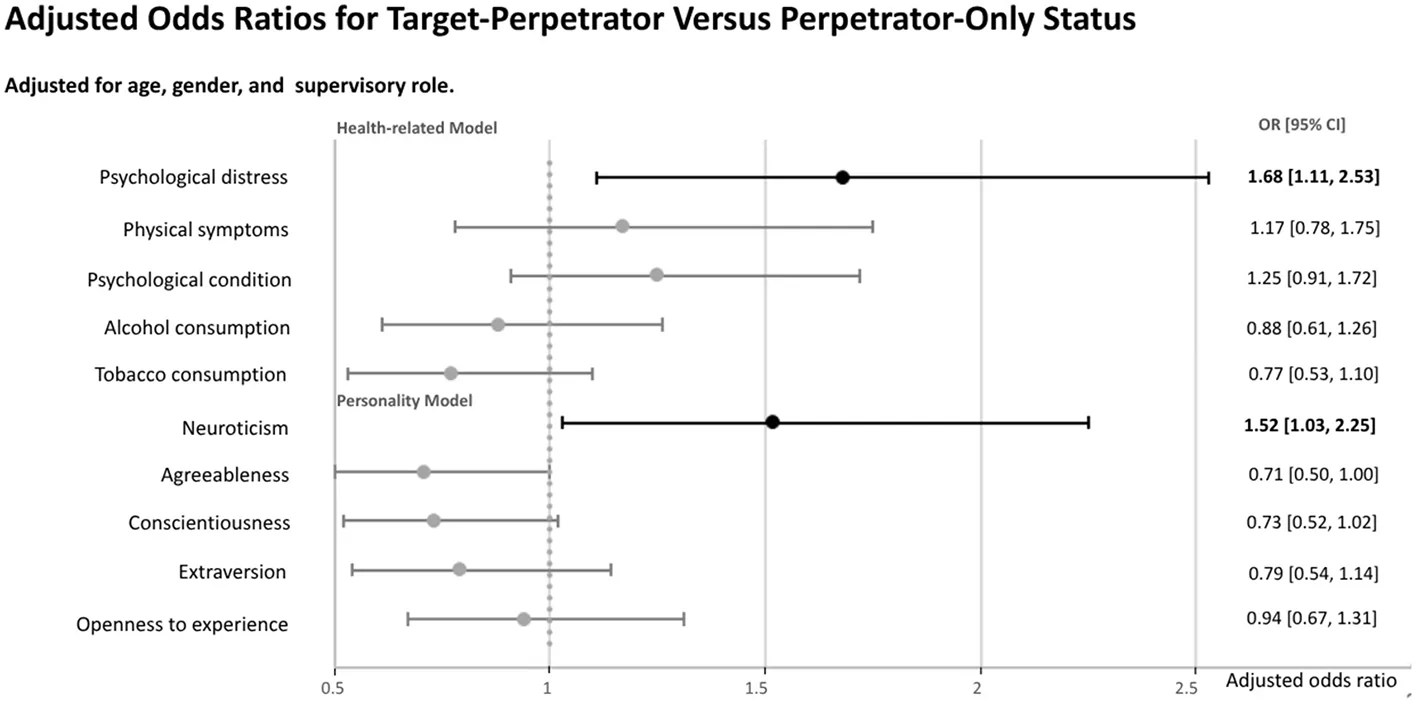
Adjusted odds ratios for target-perpetrator vs. perpetrator-only status.

Because the direct comparison between target-perpetrator and perpetrator-only groups involved a relatively small perpetrator-only cell, a stratified bootstrap robustness check was conducted to evaluate the precision and stability of the logistic regression estimates. Full bootstrap estimates are reported in Supplementary Table S1. In the adjusted health-related model, psychological distress remained a robust predictor of target-perpetrator status, *B* = 0.52, bootstrap SE = 0.24, *p* = 0.014, 95% bootstrap CI for B [0.15, 1.11]. None of the other health-related variables independently distinguished target-perpetrator from the perpetrator-only group in the bootstrap analysis. In the adjusted personality model, neuroticism remained positive but was attenuated and not significant, *B* = 0.42, bootstrap SE = 0.24, *p* = 0.052, 95% bootstrap CI for B [0.004, 0.96]. Therefore, the bootstrap analysis strengthened confidence in psychological distress as a robust predictor, whereas the neuroticism finding should be interpreted as less stable. Gender also emerged as a significant covariate in the adjusted personality bootstrap model, *B* = −0.44, bootstrap SE = 0.23, *p* = 0.037, 95% bootstrap CI for B [−0.94, −0.03]. This indicated that male employees had lower odds of belonging to the target-perpetrator group rather than the perpetrator-only group, after accounting for age, supervisory role and the personality variables. Age and supervisory role were not significant covariates. Overall, psychological distress was the clearest health-related correlate, whereas neuroticism was the clearest personality correlate distinguishing target-perpetrator from perpetrator-only group membership. Neuroticism also showed a significant positive association with target-perpetrator status in the adjusted personality model; however, this result should be interpreted cautiously given the low internal consistency of the Neuroticism subscale.

In the sensitivity analysis (Supplementary Table S2), applying the weekly-occurrence criterion substantially altered the distribution of WB roles and reduced the size of all three WB-involved groups. Of the 2,359 participants in the primary sample, 2,357 could be classified under the stricter criterion. Among these participants, 184 (7.81%) were classified as target-only, 28 (1.19%) as perpetrator-only, 22 (0.93%) as target-perpetrators, and 2,123 (90.07%) as not involved. Two participants could not be classified because of incomplete responses to the relevant WB items.

In the adjusted health-related model, psychological distress retained a positive effect estimate that was similar in magnitude to that observed in the primary analysis; however, the association was no longer statistically significant, OR = 1.79, 95% CI [0.94, 3.40]. Physical symptoms were associated with greater odds of target-perpetrator rather than perpetrator-only membership, OR = 2.45, 95% CI [1.02, 5.90], whereas tobacco consumption showed an inverse association, OR = 0.39, 95% CI [0.15, 1.00]. In the adjusted personality model, none of the focal personality traits significantly differentiated target-perpetrator from perpetrator-only membership. Supervisory role was associated with lower odds of target-perpetrator rather than perpetrator-only membership, OR = 0.45, 95% CI [0.21, 0.94].

Overall, the pattern of statistically significant associations differed across the two classification thresholds. The findings were therefore sensitive to the operationalisation of WB involvement. In addition, the weekly-occurrence criterion produced very small perpetration-related groups, resulting in wider confidence intervals and reduced precision.

## Discussion

The present study examined whether perpetrator-only and target-perpetrator groups represent distinct forms of WB involvement. Under the primary classification, the clearest difference between the two groups was in current psychological distress, which was higher among the target-perpetrator group. Evidence for broader differentiation across health-related behaviors and personality traits was limited, and the association with neuroticism was not robust to the adjusted bootstrap estimation. Overall, the findings suggest a narrow rather than global distinction between the two perpetration-related groups— and one that was itself sensitive to operationalisation: the distress difference was attenuated under the stricter weekly-occurrence classification, suggesting some sensitivity to how WB role involvement is operationalised, although the substantially smaller groups under this criterion also reduced statistical precision. Accordingly, H1a was supported under the primary classification, whereas H2a was not robustly supported and the remaining health-related and personality hypotheses were not supported.

These findings are partly consistent with previous research linking dual-role involvement to psychological distress and defensive or retaliatory responding. Earlier research suggests that some target-perpetrators may understand their own enactment of negative behaviors as a way of coping with victimization or improving their circumstances (Brotheridge et al., 2012), while diary evidence indicates that employees who experience bullying may respond defensively or retaliate in ways consistent with resource loss and self-protection (Özer et al., 2023). The higher psychological distress observed among target-perpetrators under the primary classification is consistent with this broader pattern and with previous evidence that individuals occupying both target and perpetrator roles may experience elevated mental-health risk (Özdemir and Stattin, 2011). However, the present study did not identify a broadly distinctive profile across health-related behaviors and personality traits. The findings therefore refine previous role-based research by suggesting that the distinction between target-perpetrator and perpetrator-only involvement may be concentrated in current psychological distress rather than extending consistently across dispositional and health-related domains. Moreover, the attenuation of the distress difference under the stricter weekly-occurrence criterion suggests that differences in how bullying involvement and role membership are operationalised may partly contribute to variation across studies.

Against this empirical background, this study makes three main contributions. First, it demonstrates the value of distinguishing perpetration that co-occurs with victimization from perpetration reported in the absence of victimization, rather than treating all employees who report perpetration as a homogeneous group. Although the differences observed were limited rather than comprehensive, separating these roles provides a basis for examining whether different psychological and organizational process may underlie different forms of perpetration-related involvement.

Second, the study offers a role-sensitive theoretical interpretation of perpetration-related involvement informed by Conservation of Resources theory and Social Dominance Theory. Conservation of Resources theory may be particularly useful for understanding target-perpetrator involvement as potentially embedded in resource loss, threat, and defensive or retaliatory responding. Social Dominance Theory provides a plausible lens for interpreting perpetrator-only involvement in relation to authority, control, and hierarchy-maintaining behavior. Considered together, these perspectives provide a theoretical framework for understanding heterogeneity in WB perpetration and supports the development of theory-informed, role-sensitive intervention approaches.

Third, the study has methodological and practical implications. Distinguishing perpetrator-only from target-perpetrator involvement, rather than relying solely on aggregated perpetration scores, allows potentially meaningful differences between forms of perpetration-related involvement to be examined directly. This role-sensitive approach may also help inform the development and evaluation of organizational responses tailored to the different contexts in which perpetration occurs.

### Perpetrator-only

The perpetrator-only profile may be interpreted through Social Dominance Theory, which foregrounds organizational power, control, and status maintenance. From this perspective, bullying behaviors can be interpreted as hierarchy-enhancing actions through which individuals maintain control, reinforce unequal interpersonal relations, or protect occupational position (Sidanius and Pratto, 1999). In workplaces, such behaviors may be more likely to be observed in contexts marked by supervisory authority, weak accountability, and hierarchical cultures in which intimidating or exclusionary behavior is normalized. The gender pattern observed in the present study is also compatible with accounts emphasizing power imbalance and organizational hierarchy in WB perpetration. Men are more often placed in supervisory roles and may be more likely to employ direct forms of aggression, such as shouting or public humiliation, that are often tolerated, or even reinforced, within hierarchical and male-dominated organizational cultures (Zapf et al., 2020).

Because most participants lived in Türkiye or Spain, these patterns should also be interpreted within national contexts that may shape how negative workplace behaviors are understood and reported as bullying (Salin et al., 2019). However, gender and supervisory status are structural and demographic characteristics rather than direct indicators of social dominance orientation or dominance motives. The present findings should therefore be understood as suggestive of, rather than confirming, a dominance-oriented pathway.

At the same time, the perpetrator-only group should not be viewed as unaffected or free from health-related risk. Although these variables did not directly differentiate perpetrator-only from target-perpetrator involvement, the four-group comparisons indicated that the perpetrator-only group reported higher levels of physical symptoms and tobacco consumption than the not-involved group. The tobacco finding can be interpreted within the demographic and national of the study. Because men were overrepresented in this group, gender differences and national social norms surrounding tobacco use may partly account for the observed difference (Kilic and Ozturk, 2014; East et al., 2018).

Similarly, the absence of clear differentiation by agreeableness or conscientiousness argues against explaining perpetrator-only involvement simply in terms of poor interpersonal cooperativeness or weak self-regulation. The direction of the agreeableness estimate may help inform future hypotheses, but it should not be treated as confirmatory evidence.

#### Implications

Organizational responses to perpetrator-only involvement should emphasize behavioral accountability, particularly where employees occupy supervisory positions. Because members of this group may not present with high psychological distress, organizations should not rely solely on signs of distress to identify problematic behavior. Clear behavioral standards, confidential reporting channels, and consistent investigation procedures are therefore important.

Leadership development may also be beneficial, particularly when it promotes ethical, transparent, and accountable management and addresses the power imbalances that can enable bullying (Zapf and Beitler, 2022). Conflict-resolution workshops may complement this by strengthening communication and problem-solving skills, facilitating more positive workplace interactions (Ferris et al., 2018). In addition, according to Barlett and Anderson (2011), reappraisal-based training that combines attributional reappraisal to reduce hostile attributions and interpersonal blame with cognitive reappraisal to regulate anger may help members of the perpetrator-only group interrupt the appraisal-to-aggression pathway before WB behavior is enacted. Also, evidence suggests that expressive writing can significantly reduce WB perpetration (Kirk et al., 2011).

### Target-perpetrator

Target-perpetrator involvement may be understood through a reciprocal-escalation account. Experiences of victimization, resource depletion, threat appraisal, and defensive or retaliatory behavior may become mutually reinforcing over time. For some employees, the enactment of negative acts may therefore represent a maladaptive attempt to cope with bullying exposure, restore threatened control, or protect depleted resources. This interpretation does not excuse harmful conduct; rather, it places that conduct within the broader interpersonal and organizational context in which it occurs. Because the present analysis is cross-sectional, the temporal ordering of these processes cannot be established, but the co-occurrence of victimization and elevated psychological distress provides a clear rationale for examining such cycles longitudinally.

Conservation of Resources theory offers a framework for explaining how this escalation may occur. When resources such as status, psychological safety, social support, reputation, and control are threatened, employees may attempt to protect their remaining resources or restore a sense of agency. In some cases, these attempts may take maladaptive forms, including retaliation, intimidation, exclusion, or other negative interpersonal behaviors. Such responses may provide a temporary sense of control while increasing conflict and generating further resource loss. Some employees accused of bullying may also perceive themselves as targets and justify their behavior as necessary management rather than intentional harm (Jenkins et al., 2012), which may reduce feelings of helplessness but fosters reliance on unhealthy coping behaviors, such as smoking or drinking (Nielsen et al., 2018). From a physiological perspective, hostility and anger expression are associated with low-grade inflammation and cognitive decline (See et al., 2022), as well as coronary heart disease (Miller et al., 1996). Thus, dual-role involvement may mark a subgroup with potential long-term health relevance, although longitudinal evidence would be required to evaluate temporal and causal links.

#### Implications

Target-perpetrators require interventions that address both sides of their role involvement. Organizations have a duty of care toward target-perpetrators, particularly because these individuals may experience psychological distress, ongoing psychological conditions and prior experiences of being bullied. Therefore, organizations should first create conditions that support the safe disclosure of psychological conditions and bullying experiences, reduce stigma and facilitate timely access to support. This may include mental-health inclusivity workshops, employee assistance programmes, feasible workplace accommodations, and clear pathways for employees to speak with managers or HR when they experience stress or interpersonal conflicts (Hennekam et al., 2021). Specific training programmes should also raise awareness of WB, clarify reporting procedures, and help employees recognize how stress and emotional dysregulation may increase the risk of retaliatory behavior.

Second, organizations should assess whether employees accused of bullying have also experienced bullying, while still maintaining clear boundaries around unacceptable conduct. Such assessments should not be used to excuse harmful behavior, but rather to inform interventions that address both victimization and perpetration. Purely punitive responses may therefore be insufficient because they may overlook distress, victimization, and emotional dysregulation that can contribute to behavioral escalation. Interventions should combine psychological support, conflict de-escalation, and work-design review. Leaders should review work design and implement personalized adjustments to reduce high-strain jobs characterized by imbalanced job demands and resources (Özer and Escartín, 2023). Finally, because reciprocal WB is more likely when psychosocial safety climate (PSC) is weak and prior victimization is present, organizations should strengthen PSC and account for previous levels of WB victimization to reduce escalation and prevent role entrenchment (Escartín et al., 2013).

### Differentiating target-perpetrator and perpetrator-only groups

Taken together, current psychological distress, rather than a stable personality profile, provided the clearest basis for differentiating target-perpetrator and perpetrator-only involvement under the primary classification. Although higher neuroticism initially appeared to characterize target-perpetrators, this association weakened in the adjusted bootstrap analysis and should therefore be treated as suggestive rather than confirmatory.

Conservation of Resources theory and Social Dominance Theory should therefore be viewed as complementary rather than mutually exclusive explanations. Conservation of Resources theory may be especially useful for understanding the heightened strain associated with target-perpetrator involvement. However, the perpetrator-only group may also experience resource threats involving status, reputation or social support. Maintaining or defending these resources may itself generate strain. Social Dominance Theory, in turn, provides a useful lens for interpreting perpetrator-only involvement, particularly given the over-representation of supervisors in this group and the possible roles of authority, control, and hierarchy maintenance. However, Social Dominance Theory may also help explain target-perpetrator involvement, as negative behavior may serve a status-restoring function when victimization is perceived as a threat to control or relative standing. These two theories may therefore illuminate different aspects of both forms of involvement rather than mapping exclusively onto one group. The present study did not directly measure resource loss, perceived threat, and dominance- or restoration-related motives for perpetration. The mechanisms cannot therefore be confirmed from the current findings. Future research should measure them directly and examine whether they explain transitions into different forms of perpetration-related involvement.

### Limitations and future research directions

This study has several limitations. First, the cross-sectional design precludes causal inference. Longitudinal research is needed to examine how health-related factors and personality traits develop over time across WB role groups.

Second, the use of convenience sampling limits the generalisability of the findings. Participants were recruited through social media and academic networks, and most resided in Türkiye (57.69%) and Spain (37.81%). The sample should therefore not be considered representative of the wider working populations of either country, and the findings may not generalize to other national or cultural contexts. Data were also collected during the COVID-19 pandemic, a period of elevated population-level psychological distress, which may limit the generalisability of the observed distress levels to non-pandemic contexts.

Third, the perpetrator-only group was small. Although the total analytic sample was large, the key direct comparison included only 51 perpetrator-only participants. This may partly reflect the inherent difficulty of studying WB perpetration. The behavioral-report method avoids requiring participants to self-label as perpetrators, but enactment was still assessed by self-report and may be affected by social desirability bias. This bias may not operate equally across groups. The target-perpetrator group may be more willing to disclose enactment of negative acts, because their own victimization can provide a contextual explanation or justification for their behavior (Falla et al., 2023). In contrast, the perpetrator-only group may be less willing to disclose behaviors that are stigmatized or socially undesirable (Parkins et al., 2006). This potential disclosure asymmetry may have contributed to the small perpetrator-only group and may have biased estimates comparing perpetrator-only and target-perpetrator groups.

The small perpetrator-only group also reduced the precision and stability of the regression estimates. The stratified bootstrap robustness analysis reported in Supplementary Table S1 provided additional information about estimate stability and supported the robustness of the psychological distress association. In contrast, the adjusted bootstrap estimate for neuroticism was attenuated and no longer statistically significant. The neuroticism finding should therefore be interpreted cautiously and replicated in studies with larger and more balanced perpetration-related groups. Although bootstrap analyses can help assess estimate stability, they cannot compensate for limited subgroup size.

The significant Little's MCAR tests, together with the associations between missingness and openness to experience and chronic psychological condition, suggests that the data may be missing at random (MAR) rather than missing completely at random (MCAR), although MAR cannot be established from these diagnostics alone. This does not fully support the available-case and model-specific complete-case approaches used in the present study, and the reported group comparisons and regression estimates may therefore be biased. Further replications should consider using multiple imputation or full-information maximum likelihood to address missing data.

The findings were also sensitive to the frequency threshold used to define WB role involvement. Under the weekly-occurrence criterion, both perpetration-related groups became substantially smaller, the psychological distress estimate remained positive but no longer reached statistical significance, and a different pattern of health-related predictors emerged. The personality findings were also not reproduced. Accordingly, the primary results should be interpreted as applying to the broader behavioral-involvement operationalisation used in the main analysis, rather than threshold-invariant estimates. The very small groups produced by the stricter criterion also resulted in wide confidence intervals and limited precision. Future studies should compare pre-specified frequency criteria using substantially larger perpetration-related samples.

Another concern is the reliability of the Mini-IPIP personality subscales. Although the Mini-IPIP is intentionally brief, extraversion and neuroticism showed low internal consistency in the present sample. This issue is particularly important for neuroticism because it was one of the variables distinguishing target-perpetrator from the perpetrator-only group in the adjusted model. McDonald's omega provided an even lower estimate for neuroticism than Cronbach's alpha, suggesting that this subscale may contain substantial measurement error in the present sample. Low reliability increases measurement error and may reduce the precision of the estimated association. Under classical measurement-error assumptions, such unreliability would usually attenuate associations toward the null; however, it may also increase uncertainty and instability in multivariable estimates (Carroll et al., 2006).

Finally, the study focused primarily on individual-level variables and did not fully capture interpersonal and organizational determinants, such as team conflict and PSC. Most importantly, future research should move beyond conceptualizing perpetrators as a homogeneous group and examine heterogeneity within perpetrator roles. For example, person-centered analytical approaches, such as latent class analysis, latent profile analysis, or cluster analysis, could be used to identify distinct perpetrator subgroups, including proactive, reactive, or instrumental perpetration profiles. Distinguishing between these profiles would allow researchers to identify different pathways leading to perpetration and determine whether different interventions are required. Also, role transitions over time warrant further investigation. Longitudinal designs may reveal that transitions from target to perpetrator and from perpetrator to target follow different developmental pathways and underlying mechanisms. For example, movement from target to perpetrator may be associated with retaliation, emotional dysregulation, or accumulated psychological distress, whereas movement from perpetrator to target may reflect interpersonal conflict escalation, social rejection, or organizational responses to prior bullying behavior. Understanding these distinct transition patterns may help identify whether different prevention and intervention strategies are required for different role groups.

## Conclusion

This cross-sectional study of 2,359 employees showed that the target-perpetrator and perpetrator-only groups had distinct profiles. The target-perpetrator group showed the most adverse descriptive health profile, but psychological distress was the only robust adjusted correlate of membership in this group rather than the perpetrator-only group. Neuroticism showed a positive association in the conventional model, but the adjusted bootstrap estimate was not statistically significant; the association is therefore suggestive. The perpetrator-only group was characterized more strongly by supervisory-role representation than by current emotional distress. These findings are compatible with role-sensitive interpretations informed by Conservation of Resources theory and Social Dominance Theory, although the proposed mechanisms were not directly tested. Organizations should distinguish between perpetration-related role groups and combine clear behavioral accountability with mental-health and conflict-management support tailored to the pattern of involvement.

## Data Availability

The datasets presented in this study can be found in online repositories. The names of the repository/repositories and accession number(s) can be found below: the Open Science Framework (OSF) at DOI 10.17605/OSF.IO/6WUXK.
