# Supplementary material for: Not all workplace bullying perpetrators are alike: health, personality, and occupational profiles of perpetrator-only and target-perpetrator employees
Source: Front Psychol. 2026 Sep 23;17:1890622. doi: 10.3389/fpsyg.2026.1890622 (PMC13642183; doi:10.3389/fpsyg.2026.1890622)
Supplement: Supplementary file 1 [file Table_1.docx]

**Supplementary material**

| **Table S1.**  *Stratified Bootstrap Robustness Checks for Logistic Regression Models Predicting Target-perpetrator Status* | | | | | | |
| --- | --- | --- | --- | --- | --- | --- |
|  | Model 1 | | | Model 2 | | |
|  | *B* | Bootstrap SE | 95% bootstrap CI for B | *B* | Bootstrap SE | 95% bootstrap CI for B |
| Psychological Distress | 0.55 | 0.23 | [0.19, 1.10] ** | 0.52 | 0.24 | [0.15, 1.11] * |
| Physical Symptoms | 0.24 | 0.20 | [-0.13, 0.65] | 0.18 | 0.22 | [-0.23, 0.64] |
| Psychological Condition | 0.25 | 0.23 | [-0.04, 0.65] | 0.22 | 0.22 | [-0.08, 0.64] |
| Alcohol consumption | -0.20 | 0.19 | [-0.61, 0.16] | -0.11 | 0.21 | [-0.54, 0.29] |
| Tobacco consumption | -0.28 | 0.19 | [-0.67, 0.06] | -0.26 | 0.20 | [-0.66, 0.11] |
| Age |  |  |  | 0.12 | 0.23 | [-0.30, 0.61] |
| Gender |  |  |  | -0.32 | 0.21 | [-0.77, 0.05] |
| Supervisory role |  |  |  | -0.12 | 0.19 | [-0.49, 0.24] |
|  |  |  |  |  |  |  |
|  |  |  |  |  |  |  |
|  | Model 1 | | | Model 2 | | |
|  | *B* | Bootstrap SE | 95% bootstrap CI for B | *B* | Bootstrap SE | 95% bootstrap CI for B |
| Extraversion | -0.23 | 0.19 | [-0.64, 0.12] | -0.24 | 0.21 | [-0.68, 0.14] |
| Agreeableness | -0.32 | 0.20 | [-0.74, 0.05] | -0.35 | 0.21 | [-0.81, 0.01] |
| Conscientiousness | -0.29 | 0.17 | [-0.66, 0.01] | -0.31 | 0.19 | [-0.73, 0.01] |
| Neuroticism | 0.54 | 0.22 | [0.16, 1.02] ** | 0.42 | 0.24 | [0.004, 0.96] |
| Openness to experience | -0.06 | 0.19 | [-0.44, 0.29] | -0.06 | 0.20 | [-0.46, 0.34] |
| Age |  |  |  | 0.15 | 0.23 | [-0.25, 0.68] |
| Gender |  |  |  | -0.44 | 0.23 | [-0.94, -0.03] * |
| Supervisory role |  |  |  | -0.01 | 0.20 | [-0.39, 0.38] |
| *Note.* **p* < .05. ***p* < .01. ****p* < .001  Bootstrap estimates were based on 5,000 stratified bootstrap samples, with resampling stratified by target-perpetrator versus perpetrator-only status. | | | | | | |
| Neuroticism remained positive but was not significant in the adjusted bootstrap model, bootstrap *p* = .052. | | | | | | |

| **Table S2.**  *Sensitivity Analysis of Logistic Regression Models Using Weekly-Occurrence Classification Criterion* | | | | | | |
| --- | --- | --- | --- | --- | --- | --- |
|  | Model 1 | | | Model 2 | | |
|  | *B* | OR | 95% CI for OR | *B* | OR | 95% CI for OR |
| Psychological Distress | 0.44 | 1.55 | [0.90, 2.69] | 0.58 | 1.79 | [0.94, 3.40] |
| Physical Symptoms | 0.78 | 2.19 | [1.04, 4.59] * | 0.90 | 2.45 | [1.02, 5.90] * |
| Psychological Condition | -0.09 | 0.91 | [0.55, 1.51] | -0.21 | 0.81 | [0.44, 1.52] |
| Alcohol consumption | 0.39 | 1.47 | [0.69, 3.15] | 0.37 | 1.45 | [0.57, 3.72] |
| Tobacco consumption | -0.87 | 0.42 | [0.18, 0.95] * | -0.94 | 0.39 | [0.15, 0.996] * |
| Age |  |  |  | 0.09 | 1.10 | [0.42, 2.89] |
| Gender |  |  |  | -0.82 | 0.44 | [0.18, 1.07] |
| Supervisory role |  |  |  | -0.63 | 0.53 | [0.23, 1.22] |
|  |  |  |  |  |  |  |
|  |  |  |  |  |  |  |
|  | Model 1 | | | Model 2 | | |
|  | *B* | OR | 95% CI for OR | *B* | OR | 95% CI for OR |
| Extraversion | 0.03 | 1.03 | [0.53, 1.99] | -0.28 | 0.76 | [0.34, 1.69] |
| Agreeableness | 0.45 | 1.57 | [0.91, 2.70] | 0.61 | 1.84 | [0.98, 3.46] |
| Conscientiousness | -0.27 | 0.77 | [0.42, 1.39] | -0.02 | 0.98 | [0.44, 2.21] |
| Neuroticism | 0.63 | 1.88 | [0.79, 4.48] | 0.20 | 1.22 | [0.41, 3.68] |
| Openness to experience | 0.05 | 1.05 | [0.56, 1.99] | 0.19 | 1.21 | [0.58, 2.54] |
| Age |  |  |  | -0.28 | 0.76 | [0.29, 1.98] |
| Gender |  |  |  | -0.51 | 0.60 | [0.26, 1.38] |
| Supervisory role |  |  |  | -0.81 | 0.45 | [0.21, 0.94] * |
| *Note.* **p* < .05. ***p* < .01. ****p* < .001 | | | | | | |
|  | | | | | | |
